# Video-Assisted Thoracic Surgery for Tubercular Spondylitis

**DOI:** 10.1155/2014/963497

**Published:** 2014-04-03

**Authors:** Roop Singh, Paritosh Gogna, Sanjeev Parshad, Rajender Kumar Karwasra, Parmod Kumar Karwasra, Kiranpreet Kaur

**Affiliations:** ^1^Department of Orthopaedic Surgery, Paraplegia & Rehabilitation, Pt. B.D. Sharma PGIMS, 52 / 9-J, Medical Enclave, Rohtak, Haryana 124001, India; ^2^Department of Surgery, Pt. B.D. Sharma PGIMS, Rohtak, Haryana 124001, India; ^3^Department of Anaesthesia & Critical Care, Pt. B.D. Sharma PGIMS, Rohtak, Haryana 124001, India

## Abstract

The present study evaluated the outcome of video-assisted thoracic surgery (VATS) in 9 patients (males = 6, females = 3) with clinico-radiological diagnosis of tubercular spondylitis of the dorsal spine. The mean duration of surgery was 140.88 ± 20.09 minutes, mean blood was 417.77 ± 190.90 mL, and mean duration of postoperative hospital stay was 5.77 ± 0.97 days, Seven patients had a preoperative Grade A neurological involvement, while at the time of final followup the only deficit was Grade D power in 2 patients. In patients without bone graft placement (*n* = 6), average increase in Kyphosis angle was 16°, while in patients with bone graft placement (*n* = 3) the deformity remained stationary. At the time of final follow up, fusion was achieved in all patients, the VAS score for back pain improved from a pretreatment score of 8.3 to 2, and the function assessment yielded excellent (*n* = 4) to good (*n* = 5) results. In two patients minithoracotomy had to be resorted due to extensive pleural adhesions (*n* = 1) or difficulty in placement of graft (*n* = 1). Videoassisted thoracoscopic surgery provides a safe and effective approach in the management of spinal tuberculosis. It has the advantages of decreased blood loss and post operative morbidity with minimal complications.

## 1. Introduction

Tuberculous spondylitis, which is the most common form of skeletal TB (comprising 50% of all cases) and the most serious form of tuberculous lesions in various bones and joints, is reappearing as a problem [1–5]. In the developing world spinal TB is the main cause of kyphosis; 15% of patients treated conservatively have a considerable increase in kyphotic deformity, which in 3% to 5% is more than 60°. A severe kyphotic deformity is a major cosmetic and psychological disturbance in growing child and can result in secondary cardiorespiratory problems and late-onset paraplegia [6–9].

The standard surgical method of decompression of tubercular dorsal spine is either the anterolateral extrapleural or the open transthoracic transpleural approach. Both these approaches are sufficient for adequate decompression and graft placement but are associated with significant morbidity and require a prolonged hospital stay [10]. Video-assisted thoracic surgery (VATS) has developed very rapidly in the last two decades. The use of VATS retains the advantages of anterior spinal surgery and gives a comparable result of spinal deformity correction to that of the open approaches [11]. Although the advent of video-assisted thoracoscopic surgery (VATS) has given a valuable alternative to conventional thoracotomy with minimal morbidity there have been relatively few reports of VATS used for decompression and stabilization in active tuberculosis of thoracic spine [12, 13]. We report our preliminary experience of VATS in treating tubercular spondylitis of thoracic spine and report results and difficulties associated with the procedure.

## 2. Patients and Method

We performed video-assisted thoracoscopic surgery in 9 patients (males = 6, females = 7) with tubercular spondylitis of the dorsal spine at our centre from January 2009 to December 2011. The mean age was 37.11 ± 20.55 (range: 55–88 years) and the average final followup was 32 months (range: 24 to 41 months). The clinical diagnosis was made from patient's history and thorough general physical and neurological examination. It was then correlated with plain radiography and magnetic resonance imaging (MRI). Inclusion criteria were doubtful diagnosis, severe back pain and/or radicular pain persisting after conservative treatment, neurological deficit resulting from the presence of granulation tissue, abscess or sequestrated bone or a disc fragment compressing the dura, or a paravertebral abscess under tension. Exclusion criteria were multilevel disease, concomitant cervical or lumbar lesion, pleural adhesions, and intolerance to one-lung ventilation intraoperatively. Patients were given detailed information regarding surgical procedure. Prior written informed consent was taken from each patient explaining the procedure, risks, and benefits. They were also informed that VATS can be converted into open thoracotomy in conditions like inability to tolerate one-lung ventilation or severe pleural adhesions.

The surgery was performed under general anesthesia with a double-lumen endotracheal tube inserted for ipsilateral lung collapse and single lung ventilation. A close watch on all hemodynamic and respiratory parameters was maintained. The patients were placed in the right/left lateral decubitus position, depending on the radiologic findings (i.e., bulk of abscess and caseating tissue and destruction of body) and the relevant part was draped and prepared for a standard posterolateral thoracotomy (for conversion to standard thoracotomy in circumstance of intraoperative complication or the presence of severe pleural adhesion). With selective collapse of right/left lung, the initial trocar incision (2 cm) was made usually at the fifth or sixth intercostal space (ICS) or higher along the anterior axillary line depending upon the site of lesion. An 11-mm trocar was used to introduce the operating thoracoscope and an exploratory thoracoscopy was performed. The lesion site was identified and displayed on the video monitor. Two other stab incisions, the extended manipulating channels, usually 3-4 cm in length, were done 2-3 intercostal spaces above and below the first port, slightly posterior to the posterior axillary line. We encountered difficulty in making portals due to overcrowding of ribs in two patients.

**Figure 1:**
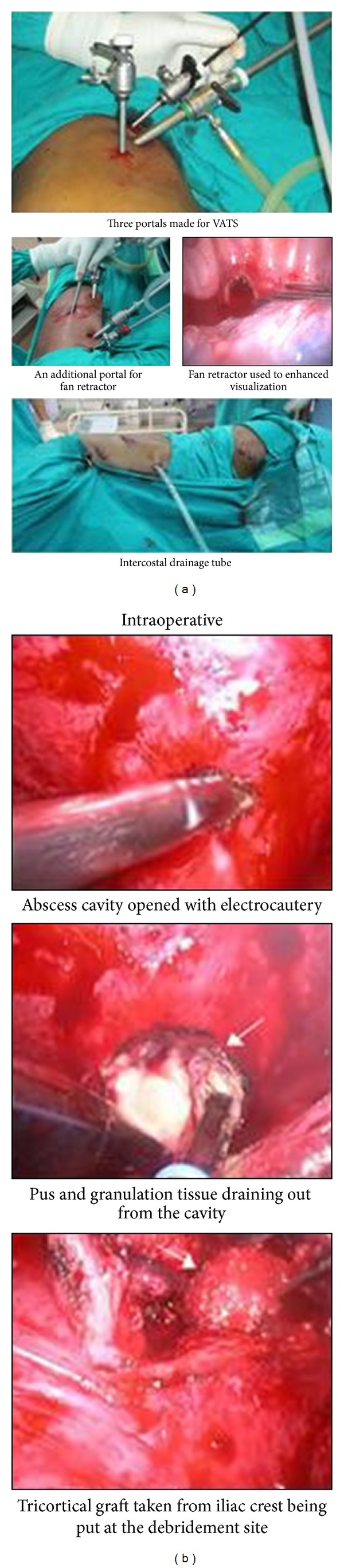
Peroperative and postoperative photographs of the VATS. (a) Portal placements and intraoperative and postoperative chest tube drainage. (b) Peroperative photographs showing opening of the lesion and debridement. Tricortical graft was put after debridement.

Visualization of the spine was enhanced by tilting the patient forward so that the collapsed lung fell anteriorly and, if required, a fan retractor for further retraction of ipsilateral lung was inserted. The correct level of diseased vertebrae was determined by counting the ribs as seen through the endoscope. Putting a spinal needle from the marker site and visualizing the tip of needle through the thoracoscope further confirmed the correct level. With monopolar electrocautery accompanied by a suction tube the parietal pleura overlying the lesion was divided longitudinally. The larger intercostal arteries and veins were isolated, ligated, and divided if needed. The biopsy and decompression procedure was then performed with conventional disc roungeurs and elongated bone curettes and was carried out down to the epidural space. Additional procedures like placement of bone graft into the intervertebral space using a conventional bone impactor were done in 3 patients. In 2 patients, conversion to minithoracotomy was undertaken. A larger manipulating channel measuring 5 to 6 cm in length was created on the right/left lateral chest after introducing the thoracoscope and a short-segment rib of equal length was removed. The incision was made slightly behind the posterior axillary line at the level of right fifth rib. Then a rib spreader was used to open the intercostal space. Adhesionolysis by blunt dissection using finger was done. The following spinal procedures, including debridement, sequesterectomy, and interbody fusion with tricortical iliac crest bone grafting, could be manipulated with techniques used for standard open surgical procedures. The material was sent for histopathology, culture, and Ziehl-Neelsen staining. Hemostasis was carefully monitored and chest tube drain of appropriate size was inserted through one of the port sites in 7th or 8th intercostal space in midaxillary line and was connected to an underwater seal. The small wounds were closed.  Figure 1 shows the various steps of VATS viz. placements of portal, fan retractor, chest tube drainage, opening abscess cavity and graft.

Patients were kept under close observation for 24 hours. A plain radiograph of the chest was obtained for adequate lung inflation. Chest tube was removed once collection in the chest tube bag was <50 mL in 24 hours. Postoperative X-rays were taken to assess the improvement. Stitches were removed after two weeks. Patient was advised bed rest for a minimal of 6 weeks. Mobilization was started after 6 weeks using a thoracolumbosacral orthosis (TLSO)/modified Taylor's brace with axillary support depending upon the clinical status of patient. ATT was given for 12 months. Patients were followed up at 2 weeks for 1 month, monthly for the next 6 months, and thereafter once in every 3 months. At each followup patient was examined clinicoradiologically and laboratory investigations (complete blood haemogram, serum glutamic oxaloacetic transaminase/serum glutamic pyruvic transaminase, serum bilirubin, serum protein, and albumin/globulin ratio) were done. At the time of final followup MRI and computed tomography (CT scan) of the dorsal spine were also performed.

The surgical outcome was assessed in terms of preoperative and postoperative neurologic status as per Frankel's grading, operative time, blood loss, average hospital stay, deformity correction and maintenance, fusion status, back pain using visual analogue scale, and complications. Fusion was assessed using both plain radiographs and CT scan and using Eck et al. criteria for fusion assessment [14]. Final functional outcome was assessed by modified Kirkaldy-Willis criteria [18].

## 3. Results

Patients were suffering from the symptomatology of the TB with a mean duration of 8.44 ± 3 months (range: 5–12 months). All the patients (*n* = 09) received antituberculous treatment (ATT) for a period of 3-4 weeks minimum before surgery and then postoperatively. The total duration of ATT was 12 months. The indication for surgery using VATS was failure to respond to chemotherapy (*n* = 01), neurological deficit not responding to chemotherapy (*n* = 07), and doubtful diagnosis (*n* = 01).

Using VATS, debridement, drainage of prevertebral and paravertebral abscess, and decompression of cord were done in six patients; debridement, drainage, decompression, and reconstruction with bone graft were done in one patient; and debridement, drainage, decompression, reconstruction with bone graft, and minithoracotomy were done in two patients. Sufficient tissue for histopathological examination was obtained and the clinical diagnosis of tuberculosis of spine was confirmed by pathologists in all the cases. The average operative time was 140.88 ± 20.09 minutes (range: 105–165 minutes), average blood loss was 417.77 ± 190.90 mL (range: 220–730 mL), and average hospital stay was 5.77 ± 0.97 days (range: 4–7 days). As per Frankel's grading, 7 patients had Grade A neurological involvement preoperatively, which improved at subsequent followups (Table 1).

Radiographs of the spine revealed wedge collapse with contagious involvement in all patients. Average vertebral height loss, deformity angle, and kyphotic angle initially were 0.48, 11.8°, and 24.2°, respectively; the final values were 01, 22°, and 37°, respectively. As per CT the average percentage canal encroachment was 52.7% at initial presentation which improved to 10% at the time of final followup; it also revealed that fusion was present in 75% of the patients at their final followup. On MRI, all patients showed paradiscal and contiguous involvement of vertebrae; average vertebrae involvement per patient was 2.88 at presentation and 2.33 at the time of final followup. Paravertebral collection and subligamentous spread were seen in all patients at initial presentation, with an average vertebral extent of paravertebral soft tissue collection and subligamentous spread as 4.3 vertebrae each initially, which dropped to 2.7 and 1 vertebrae, respectively, at time of final followup.

The mean preoperative kyphosis angle in patient without (*n* = 6) and with (*n* = 3) bone graft was 25° and 23° and at time of final followup was 41° and 24°, respectively. Two of the six patients without bone grafting achieved fusion at six months and another four at 12 months. Eck et al. criteria for fusion assessment were used to grade the fusion in 3 patients with bone grafting, according to which all 3 cases achieved Grade II fusion at six months and showed further improvement to Grade I at 12 months (Table 2).

Back pain as assessed using visual analogue scale improved from a pretreatment score of 8.3 to 2 at final followup. Functional outcome assessed as per the modified Kirkaldy-Willis criteria revealed 3 patients to have an excellent outcome, while good outcome was observed in 1 patient.

**Figure 2:**
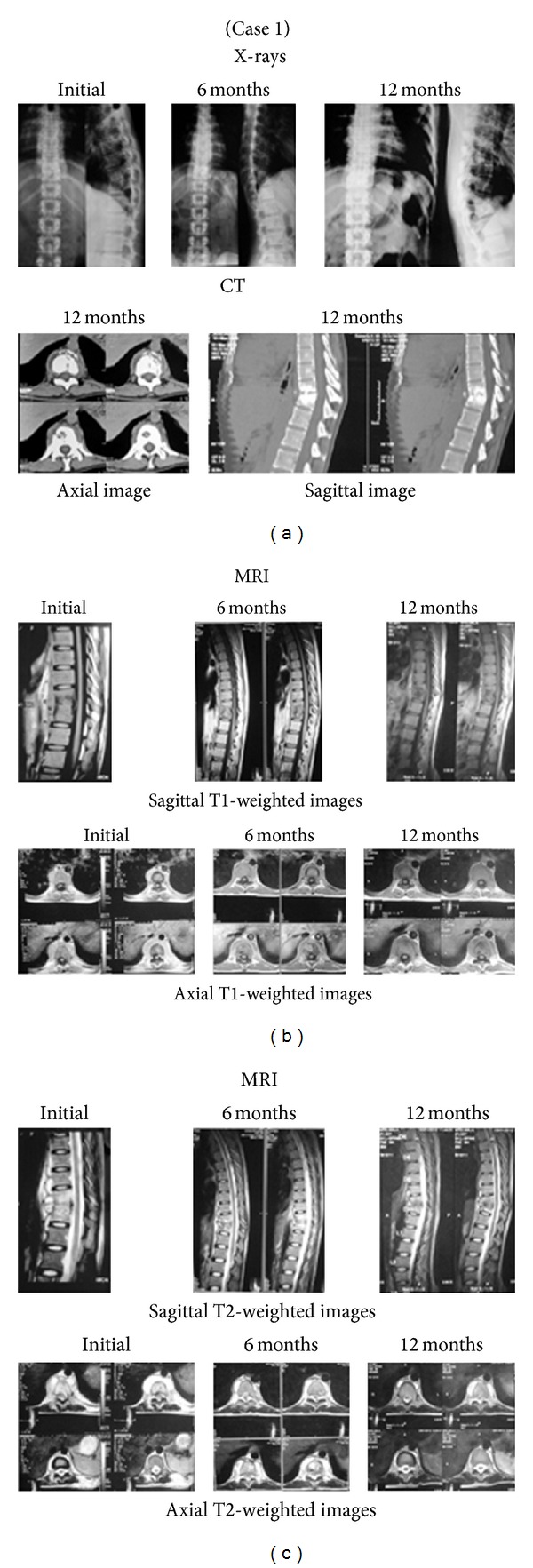
Tubercular spondylitis thoracic spine (D10-D11). Patient had a good subjective outcome and all changes in laboratory and radiological (MRI, CT, and X-rays) parameters showed improvement by the end of 12 months. Fusion was achieved at 12 months. No complications were seen and sinus which was present at initial presentation completely healed at her 18th month followup. (a) shows initial, 6-month, and 12-month X-rays and CT scan of the patient. (b) shows initial, 6-month, and 12-month sagittal and axial T1-weighted images. Disease completely healed at 12 months. (c) shows initial, 6-month, and 12-month sagittal and axial T2-weighted images. Disease completely healed at 12 months.

**Figure 3:**
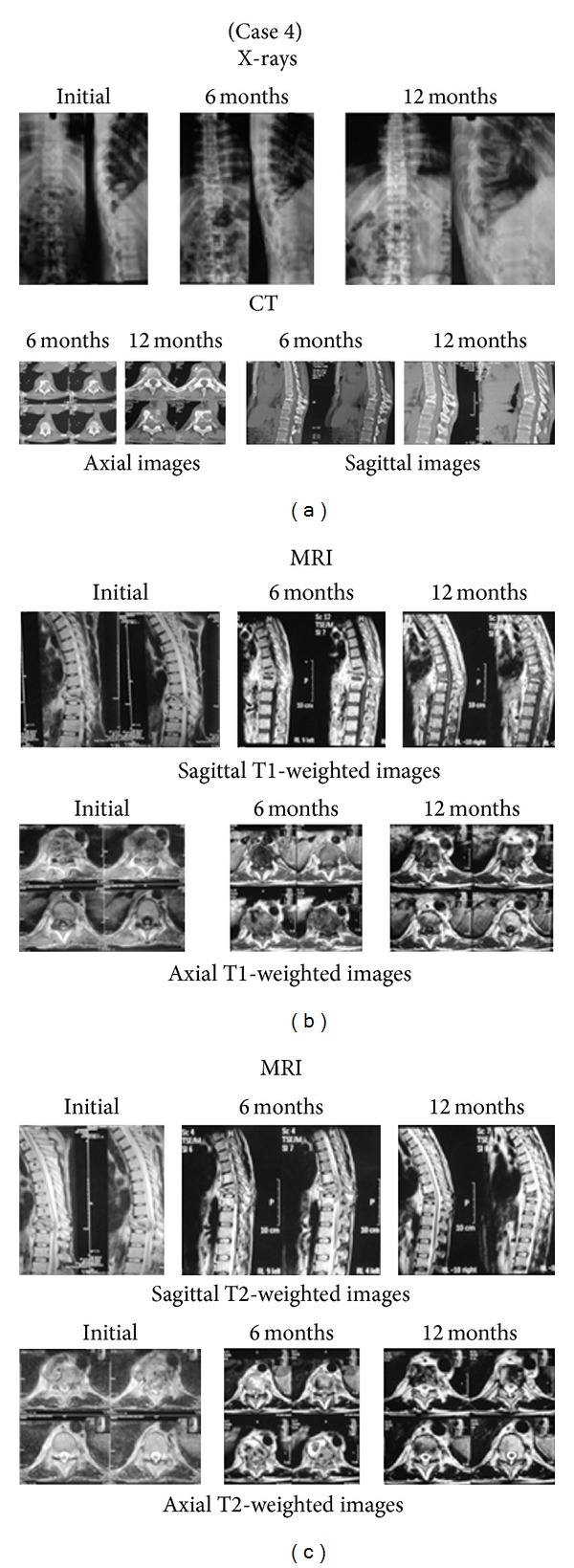
Tubercular spondylitisthoracic (D9-D10) with neurologic deficit. VATS along with minithoracotomy and placement of bone graft was done. Conversion to minithoracotomy was done because of dense pleural adhesions and difficulty in making portals was also encountered. Excellent subjective outcome and improvement in all laboratory and radiological (MRI, CT, and X-rays) parameters was seen by the end of 12 months. Patient attained ambulatory power (Grade A neurological deficit preoperatively) within 6 months of surgery. Grade I fusion was achieved at 12 months. (a) shows initial, 6-month, and 12-month X-rays and CT scan of the patient. (b) shows initial, 6-month, and 12-month sagittal and axial T1-weighted images. Disease completely healed at 12 months. (c) shows initial, 6-month, and 12-month sagittal and axial T2-weighted images. Disease completely healed at 12 months.

The most common complication was conversion to minithoracotomy in two patients. It was due to extensive pleural adhesions leading to difficulty in graft placement in one case and bleeding during placement of portals in another case. None of our cases had pneumothorax, pneumonitis, chylothorax, or Horner's syndrome. Postoperative histopathological examination showed caseation and/or granuloma formation suggestive of tuberculosis in all cases. Figures 2 and 3 are the photographs of X-rays, CT and MRI of the representative cases at presentation, six months, and 12 months.

## 4. Discussion

Evidence of tuberculous spondylitis, probably due to infection with mycobacterium bovis, was identified in mummies from the tomb of nebeveenenf, indicating that this process existed in dynastic Egypt as early as 3700 BC [19]. Skeletal tuberculosis still remains a major health concern as it accounts for at least 10% of cases of extrapulmonary infection, and spine is the most common site of bony involvement [10].

Absolute indications for surgery in patients with spinal tuberculosis under active treatment are approximately 6% in those without neurologic deficit and approximately 60% in those with neurologic deficit [20]. The standard surgical method of decompression of tubercular dorsal spine is either the anterolateral extrapleural or open transthoracic transpleural approach. Both these approaches are sufficient for adequate decompression and graft placement but are associated with significant morbidity and require a prolonged hospital stay [15]. Video-assisted thoracoscopic surgery (VATS) is a good surgical alternative to conventional thoracotomy with minimal morbidity [21], though surgically demanding. VATS has been used extensively in spinal deformities such as scoliosis with results comparable to open procedures, but there has been limited use of VATS for decompression in active tuberculosis of dorsal spine [16].

It is recommended to do bone grafting in tuberculous spine when significant bone loss has occurred. Once the adjacent vertebral bodies develop destructive lesions, vertebral collapse may follow, due to destruction of cancellous bone, producing anterior or lateral wedging. Bone graft provides the stability and prevents further collapse of spine [12]. In our study, bone grafting was done in three patients.

The operative time in our series ranged from 105 to 165 minutes, this variation was due to the different types of procedures performed, and, as expected, the operative time for each procedure was longer initially and decreased with experience. Our mean operative time was less as compared to other studies (Table 3) because we did not go for spinal instrumentation and bone grafting was done only in three patients in this short series. Average blood loss increases with the operative time and addition of an additional procedure. Better view provided by thoracoscopy and its preservation of wall structures (less extensive tissue dissection) probably are the explanations for less bleeding. Blood loss was comparative to other series of VATS in tuberculosis spine [13, 15, 16], except studies by Jayaswal et al. [12] and Kandwal et al. [17] where they used spinal instrumentation for stabilization in addition to the debridement. One of the major reported advantages of VATS was the reduction in postoperative hospital stay, and this was also observed in our series [22, 23]. Postoperative stay was less than reported by thoracotomy patients in other studies [23], which is a major consideration in developing countries with a high patient load in tertiary care hospitals.

One of the major goals of surgery was to achieve adequate neurological decompression through VATS in the present study. The decompression was adequate as indicated by the neurological recovery in all our cases. Our results are in accordance with available literature showing neurological recovery varying from 82 to 95% recovery of ambulatory status [12, 13, 15–17]. In a retrospective study done by Jayaswal et al. (2007), postoperatively 17 of the 18 patients with preoperative neurologic deficit attained ambulatory status and all patients showed improvement on the Frankel scale, with Grade C in one patient, Grade D in 10 patients, and Grade E in 12 patients [12]. In a series by Kapoor et al. (2005) of 16 patients, 14 (88%) had good neurologic recovery (improvement by 2-3 grades). In one patient, thoracoscopy was abandoned, and open thoracotomy was performed. Another patient did not recover and underwent anterolateral decompression after 10 weeks [16]. In another series of 30 patients by Kapoor et al. (2012), all patients improved neurologically on a mean followup of 80 months. No patient had neurological deterioration and all of them regained ambulatory power with no cases of recurrence of tuberculosis [13]. In a series by Huang et al. (2000), after a followup of 24 months, the average neurologic recovery was 1.1 grades on Frankel's scale [15].

In our study, the mean preoperative, postoperative, 6-month, and 12-month kyphosis angle in patients without bone graft placement were 25°, 32°, and 41°, respectively. Therefore, final X-ray examination revealed an average increase in kyphosis angle by 16°. The mean preoperative, postoperative, 6-month, and 12-month kyphosis angle in patients with bone graft placement were 23°, 18°, and 24°, respectively. Therefore, there is an initial decrease in kyphosis angle with a subsequent slight increase at final followup, with deformity remaining stationary in the patients where bone grafting was done. Similar results were obtained by Jayaswal et al. (2007) with mean preoperative and 12-month follow-up kyphosis angles being 28° and 32° in patients without bone graft placement. The mean preoperative and 12-month follow-up kyphosis angles were 34° and 31° in patients in whom reconstruction with bone graft was done [12]. In a series by Huang et al. (2000), the mean preoperative, postoperative, and 2-year follow-up kyphosis angles were 26.8°, 16.8°, and 26°, respectively [15].

Adequate debridement and decompression also make room for healthy cancellous bone apposition resulting in high fusion rates [24, 25]. In a series of 23 patients who underwent VATS by Jayaswal et al. (2007), 22 achieved fusion with an average time for fusion of 16.5 weeks. Sixteen patients had Grade I fusion and six had Grade II fusion, and failure of fusion was seen in one patient [12]. In a series by Kandwal et al. (2012), 22 of 23 patients who underwent VATS had good fusion (Grade I and Grade II) and there was failure of fusion in one patient [17]. All our cases were able to attain fusion; this slight variation from that of literature can be attributable to small sample size of our study.

In our study, the VAS score for back pain improved from a pretreatment score of 8.3 to posttreatment 6-month and 12-month scores of 3.3 and 2, respectively. Kapoor et al. (2012) reported a statistically significant difference (*P* < 0.001 with Student's *t*-test) in VAS for back pain at three months compared to the preoperative period and at five-year followup compared to three months (*P* < 0.001) [13].

Functional outcome as assessed by modified Kirkaldy-Willis criteria at the time of final followup revealed result to be either excellent (*n* = 5) or good (*n* = 4). Huang et al. (2000) in their study of 10 patients followed for 24 months reported results as excellent (*n* = 4), good (*n* = 5), or fair (*n* = 1) [15]. In a series by Kapoor et al. (2012), out of 30 patients, excellent results were obtained in 24 patients, good in four, and fair in two, with 95% of patients having a good or excellent result [13].

As far as complications are concerned, all the complications of conventional thoracotomy are possible with the VATS procedure with a reported rate of 24.4–31.3% [16]. Dense pleural adhesion was encountered in two patients and to complete the procedure, we had to convert VATS into minithoracotomy. This has been reported as a complication of the procedure by others [12, 13, 15]. But we believe that this is not a complication of VATS per se, but a limitation of the procedure. None of the patients had intercostal neuralgia, which is a common complication in video-assisted thoracoscopic surgery (VATS). We did not encounter other complications of VATS reported in the literature like wound infection, dural tear, increase in neurologic deficit, chylothorax, Horner syndrome, encysted effusion, postoperative air leak, pneumothorax [26].

Our study has its own set of limitations. To name them, the study population was small and control group was lacking. Also, this series describes our early experiences with VATS. There is a steep learning curve before all the surgical goals of the open method can be attained through VATS. Adequate hand and eye coordination, which is necessary to perform remote bone and soft tissue dissection and to establish proper orientation under the angled endoscope, is required [12, 13, 15–17]. However, the strength of the study is that it is a single institutional study with cases operated by the same team of surgeons. The fact that this study comes from a centre in peripheral area of a developing country, where the prevalence of TB spondylitis is high, further adds to the relevance of this study.

To conclude, anterolateral decompression and transthoracic anterior decompression have been the two favoured approaches, but VATS can be considered as a valuable adjunct to the available options in the modern era of minimally invasive spine surgery. The findings of the present study suggest that video-assisted thoracoscopic surgery provides a safe and effective approach to the diagnosis and management of spinal tuberculosis.

It has inherent advantages of decreased blood loss and postoperative morbidity with good cosmetic acceptance but requires a learning curve and proper armamentarium. Proper selection of patients; competence of the anesthesiologist for monitoring single lung anesthesia; and surgical skills and experience of the surgeon comes handy in achieving ultimate good outcome. VATS leads to early recovery, cost effectivity, less morbidity, and shorter hospital stay. Our early experience of VATS in treating TB spondylitis is quiet encouraging and adds to the growing body in favour of minimally invasive surgery for the management of these lesions, though randomised studies with a larger followup are required to further support this observation.

## Figures and Tables

**Table 1:** Neurological improvement as per Frankel's grading.

Frankel's grades	Number of patients						
	Preop	Immediate postop	1 M	3 M	6 M	12 M	FFU
A	7	4	3	—	—	—	
B	—	1	2	2	—	—	
C	—	—	—	—	2	—	
D	—	2	2	3	3	3	2
E	2	2	2	4	4	6	7

M: months; FFU: final followup.

**Table 2:** Status of fusion in patients.

	Preop	6 months	12 months	Final followup
Fusion				
Without bone graft (*n* = 06)				
X-ray	—	04	06	06
C T		04	06	06
With bone graft (Eck et al. [14] grading) (*n* = 03)				
X-ray	—	03 (Grade II)	03 (Grade I)	03
CT	—	03 (Grade II)	03 (Grade I)	03

**Table 3:** Comparison of mean duration of surgery, average blood loss, and postoperative hospital stay with other studies.

Authors	Mean duration of surgery (minutes)	Average blood loss (mL)	Postoperative hospital stay (days)
Huang et al. (2000) [15]	172 (120–240)	485 (150–850)	21 (9–52)
Kapoor et al. (2005) [16]	223 (150–320)	497 (200–1200)	5.5 (4–9)
Jayaswal et al. (2007) [12]	228 (102–324)	780 (330–1180)	6 (3–12)
Kapoor et al. (2012) [13]	158.8	296.7	8.8
Kandwal et al. (2012) [17]	228 (102–330)	780 (330–1180)	—
Present study (2014)	141 (105–165)	418 (220–730)	5.7 (4–7)
